# Publication guidelines for quality improvement in health care: evolution of the SQUIRE project

**DOI:** 10.1136/qshc.2008.029066

**Published:** 2008-09-26

**Authors:** F Davidoff, P Batalden, D Stevens, G Ogrinc, S Mooney, for the SQUIRE development group

**Affiliations:** 1 Institute for Healthcare Improvement, New Hampshire, USA; 2 Dartmouth Institute for Health Policy and Clinical Practice, Center for Leadership and Improvement, Dartmouth College, Hanover, New Hampshire, USA

## Abstract

In 2005, draft guidelines were published for reporting studies of quality improvement interventions as the initial step in a consensus process for development of a more definitive version. This article contains the full revised version of the guidelines, which the authors refer to as SQUIRE (Standards for QUality Improvement Reporting Excellence). This paper also describes the consensus process, which included informal feedback from authors, editors and peer reviewers who used the guidelines; formal written commentaries; input from a group of publication guideline developers; ongoing review of the literature on the epistemology of improvement and methods for evaluating complex social programmes; a two-day meeting of stakeholders for critical discussion and debate of the guidelines’ content and wording; and commentary on sequential versions of the guidelines from an expert consultant group. Finally, the authors consider the major differences between SQUIRE and the initial draft guidelines; limitations of and unresolved questions about SQUIRE; ancillary supporting documents and alternative versions that are under development; and plans for dissemination, testing and further development of SQUIRE.

A great deal of meaningful and effective work is now done in clinical settings to improve the quality and safety of care. Unfortunately, relatively little of that work is reported in the biomedical literature, and much of what is published could be more effectively presented. Failure to publish is potentially a serious barrier to the development of improvement in health and medical care and improvement science generally, since public sharing of concepts, methods and findings is essential to the progress of all scientific work, both theoretical and applied. In an effort to strengthen the evidence base for improvement in health care, in 2005 we proposed draft guidelines for reporting planned original studies of improvement interventions.^1^ Our ultimate aims were to stimulate the publication of high-calibre improvement studies, and to increase the completeness, accuracy and transparency of published reports of that work.

Inadequate reporting of work in clinical improvement has been documented in several ways.^2^ ^3^ For example, a recent meta-regression analysis of improvement strategies for diabetes control concluded that “complexity of the intervention … compounded by the paucity of descriptive detail in most studies, may have resulted in misclassification of interventions”.^3^ Moreover, an informal study carried out following publication of the draft guidelines found that only 12–68% of 30 published original reports of quality improvement projects provided full information on the individual guideline items we had identified,^1^ while individual guideline items were not addressed at all in 4–44% of those reports (Mooney S, Ogrinc G, unpublished).

Our initial draft guidelines were, of course, not a tested approach to judging the quality of the improvement literature, since that draft was based largely on the authors’ personal experience with improvement work, and was intended only as an initial step towards an established standard. We have now refined and extended that draft, as planned. In the current article we present a revised version, which we refer to as the Standard for QUality Improvement Reporting Excellence, or SQUIRE (table 1). We also describe the SQUIRE consensus development process; the major differences between the current version of SQUIRE and the initial draft guidelines; limitations of and questions about SQUIRE; ancillary supporting documents and variant versions that are under development; and plans for dissemination, testing and further development of the SQUIRE guidelines.

**Table 1:** SQUIRE guidelines (Standards for QUality Improvement Reporting Excellence)*

Text section; item number and name	Section or item description
Title and abstract	Did you provide clear and accurate information for finding, indexing, and scanning your paper?
1 Title	(a) Indicates the article concerns the improvement of quality (broadly defined to include the safety, effectiveness, patient-centredness, timeliness, efficiency and equity of care)
	(b) States the specific aim of the intervention
	(c) Specifies the study method used (for example, “A qualitative study,” or “A randomised cluster trial”)
2 Abstract	Summarises precisely all key information from various sections of the text using the abstract format of the intended publication
	
Introduction	Why did you start?
3 Background knowledge	Provides a brief, non-selective summary of current knowledge of the care problem being addressed, and characteristics of organisations in which it occurs
4 Local problem	Describes the nature and severity of the specific local problem or system dysfunction that was addressed
5 Intended improvement	(a) Describes the specific aim (changes/improvements in care processes and patient outcomes) of the proposed intervention
	(b) Specifies who (champions, supporters) and what (events, observations) triggered the decision to make changes, and why now (timing)
6 Study question	States precisely the primary improvement-related question and any secondary questions that the study of the intervention was designed to answer
	
Methods	What did you do?
7 Ethical issues	Describes ethical aspects of implementing and studying the improvement, such as privacy concerns, protection of participants’ physical wellbeing and potential author conflicts of interest, and how ethical concerns were addressed
8 Setting	Specifies how elements of the local care environment considered most likely to influence change/improvement in the involved site or sites were identified and characterised
9 Planning the intervention	(a) Describes the intervention and its component parts in sufficient detail that others could reproduce it
	(b) Indicates main factors that contributed to choice of the specific intervention (for example, analysis of causes of dysfunction; matching relevant improvement experience of others with the local situation)
	(c) Outlines initial plans for how the intervention was to be implemented—for example, what was to be done (initial steps; functions to be accomplished by those steps; how tests of change would be used to modify intervention) and by whom (intended roles, qualifications, and training of staff)
10 Planning the study of the intervention	(a) Outlines plans for assessing how well the intervention was implemented (dose or intensity of exposure)
	(b) Describes mechanisms by which intervention components were expected to cause changes, and plans for testing whether those mechanisms were effective
	(c) Identifies the study design (for example, observational, quasi-experimental, experimental) chosen for measuring impact of the intervention on primary and secondary outcomes, if applicable
	(d) Explains plans for implementing essential aspects of the chosen study design, as described in publication guidelines for specific designs, if applicable (see, for example, www.equator-network.org)
	(e) Describes aspects of the study design that specifically concerned internal validity (integrity of the data) and external validity (generalisability)
11 Methods of evaluation	(a) Describes instruments and procedures (qualitative, quantitative or mixed) used to assess (a) the effectiveness of implementation, (b) the contributions of intervention components and context factors to effectiveness of the intervention and (c) primary and secondary outcomes
	(b) Reports efforts to validate and test reliability of assessment instruments
	(c) Explains methods used to assure data quality and adequacy (for example, blinding; repeating measurements and data extraction; training in data collection; collection of sufficient baseline measurements)
12 Analysis	(a) Provides details of qualitative and quantitative (statistical) methods used to draw inferences from the data
	(b) Aligns unit of analysis with level at which the intervention was implemented, if applicable
	(c) Specifies degree of variability expected in implementation, change expected in primary outcome (effect size) and ability of study design (including size) to detect such effects
	(d) Describes analytical methods used to demonstrate effects of time as a variable (for example, statistical process control)
	
Results	What did you find?
13 Outcomes	(a) Nature of setting and improvement intervention
	(i) Characterises relevant elements of setting or settings (for example, geography, physical resources, organisational culture, history of change efforts) and structures and patterns of care (for example, staffing, leadership) that provided context for the intervention
	(ii) Explains the actual course of the intervention (for example, sequence of steps, events or phases; type and number of participants at key points), preferably using a time-line diagram or flow chart
	(iii) Documents degree of success in implementing intervention components
	(iv) Describes how and why the initial plan evolved, and the most important lessons learned from that evolution, particularly the effects of internal feedback from tests of change (reflexiveness)
	(b) Changes in processes of care and patient outcomes associated with the intervention
	(i) Presents data on changes observed in the care delivery process
	(ii) Presents data on changes observed in measures of patient outcome (for example, morbidity, mortality, function, patient/staff satisfaction, service utilisation, cost, care disparities)
	(iii) Considers benefits, harms, unexpected results, problems, failures
	(iv) Presents evidence regarding the strength of association between observed changes/improvements and intervention components/context factors
	(v) Includes summary of missing data for intervention and outcomes
	
Discussion	What do the findings mean?
14 Summary	(a) Summarises the most important successes and difficulties in implementing intervention components, and main changes observed in care delivery and clinical outcomes
	(b) Highlights the study’s particular strengths
15 Relation to other evidence	Compares and contrasts study results with relevant findings of others, drawing on broad review of the literature; use of a summary table may be helpful in building on existing evidence
16 Limitations	(a) Considers possible sources of confounding, bias or imprecision in design, measurement, and analysis that might have affected study outcomes (internal validity)
	(b) Explores factors that could affect generalisability (external validity)—for example, representativeness of participants; effectiveness of implementation; dose-response effects; features of local care setting
	(c) Addresses likelihood that observed gains may weaken over time, and describes plans, if any, for monitoring and maintaining improvement; explicitly states if such planning was not done
	(d) Reviews efforts made to minimise and adjust for study limitations
	(e) Assesses the effect of study limitations on interpretation and application of results
17 Interpretation	(a) Explores possible reasons for differences between observed and expected outcomes
	(b) Draws inferences consistent with the strength of the data about causal mechanisms and size of observed changes, paying particular attention to components of the intervention and context factors that helped determine the intervention’s effectiveness (or lack thereof) and types of settings in which this intervention is most likely to be effective
	(c) Suggests steps that might be modified to improve future performance
	(d) Reviews issues of opportunity cost and actual financial cost of the intervention
18 Conclusions	(a) Considers overall practical usefulness of the intervention
	(b) Suggests implications of this report for further studies of improvement interventions
	
Other information	Were there other factors relevant to the conduct and interpretation of the study?
19 Funding	Describes funding sources, if any, and role of funding organisation in design, implementation, interpretation and publication of study

*These guidelines provide a framework for reporting formal, planned studies designed to assess the nature and effectiveness of interventions to improve the quality and safety of care. It may not always be appropriate or even possible to include information about every numbered guideline item in reports of original studies, but authors should at least consider every item in writing their reports. Although each major section (that is, Introduction, Methods, Results and Discussion) of a published original study generally contains some information about the numbered items within that section, information about items from one section (for example, the Introduction) is also often needed in other sections (for example, the Discussion).

## THE CONSENSUS PROCESS

The SQUIRE development process proceeded along six general lines. First, we obtained informal feedback on the utility, strengths and limitations of the initial draft guidelines by using them in seminars with potential authors in both the United States and other countries; others have gathered similar information from journal reviewers.^4^ We obtained additional comment at the organisational meeting of the EQUATOR Network, a group of experienced publication guideline developers, in June 2006.^5^ Second, journal editors and authors “road tested” the draft guidelines as an aid to writing and editing submitted manuscripts. In that connection, at the time of writing this paper the 2005 article^1^ has been cited in approximately 40 subsequent publications, and full text or PDF copies have been downloaded from the journal website over 15 000 times. Third, we solicited formal commentaries by several knowledgeable authors on the initial version of the guidelines.^6^^–^10 Fourth, we conducted an ongoing review of the relevant literature on epistemology, methodology and evaluation of complex interventions, particularly in social sciences and the evaluation of social programmes. Fifth, in April 2007 we subjected the draft guidelines to intensive analysis, comment and recommendations for change at a two-day meeting of 30 stakeholders. Finally, following that meeting, we obtained further critical assessment and suggestions through three cycles of a Delphi process involving an international group of more than 50 consultants.

### Informal feedback

Informal input about the draft guidelines from authors and peer reviewers generally recognised their practical value, in comments such as “These guidelines … can guide the writing of the article, so it may be prudent to distribute [them] to the authors of papers,” and “I … have used them and find them most helpful”.^4^ These users also raised several relevant issues, including (1) uncertainty as to when (that is, to which studies) the guidelines apply, (2) the possibility their use might force QI reports into a rigid, narrow format, (3) the concern that their slavish application might result in unduly lengthy reports that were indiscriminately laden with detail and (4) difficulty for authors in knowing whether, and how, other guidelines (such as the CONSORT guidelines for reporting randomised trials) should be used in conjunction with guidelines for reporting studies of improvement and safety.

#### Deciding when to use the guidelines

Publications on improvement in health care appear to be emerging in four general categories: empirical studies on development and testing of quality improvement interventions; stories, theories and frameworks; literature reviews and syntheses; and the development and testing of improvement-related tools (Rubenstein L, *et al*, unpublished). Within that context, our consensus process has made it clear that the SQUIRE guidelines can and should apply to reporting in the first category: formal planned empirical studies on the development and testing of improvement interventions.

#### Forcing articles into a rigid format

Publication guidelines are often referred to as checklists, since they serve the function of “aides-memoires” whose value in managing information in highly complex systems is increasingly appreciated.^11^ Like all constraints, checklists can of course be rigidly applied, a form of misuse that prevents rather than helps to make sense of what is being reported.^12^ ^13^ Paradoxically, constraints also serve as a crucial driver for creativity; as stated by the 19th century English writer and designer William Morris: “You can’t have art without resistance in the materials”. The SQUIRE guidelines, like all form and structure, must therefore always be understood and used as signposts, not shackles.^14^ This caution probably translates best into practice if authors simply keep the guidelines in mind as a general framework while writing their initial draft, and then use them for detailed critical appraisal of what they’ve written while they revise the text.

#### Creating longer articles

Improvement is a complex undertaking, and its evaluation can produce substantial amounts of qualitative and quantitive information. Added length can therefore meet a principal aim of SQUIRE if it makes reports of improvement studies more complete, coherent, usable, and systematic; of course, adding irrelevant information simply to “cover” guideline items distorts that purpose. Publishing portions of improvement studies electronically is one important way to make the content of long papers publicly available while preserving the scarce resource of print publication.

#### Conjoint use with other publication guidelines

Most other biomedical publication guidelines apply to the reporting of specific study designs (for example, randomised trials or observational studies). The SQUIRE guidelines, in contrast, are concerned with reporting studies in a defined content area—improvement and safety. The two guideline types are therefore complementary; when appropriate, other specific design-related guidelines can and should therefore be used in conjunction with SQUIRE.

### Road testing

The editors of the journal *Quality and Safety in Health Care* gained experience with the initial draft guidelines by using them to help judge the completeness and transparency of submitted manuscripts, and encouraging their use by authors in revising their papers.^15^^–^18 The guidelines were also used by authors participating in the Academy for Healthcare Improvement’s 2007 scientific symposium in preparing their abstracts for subsequent publication.

### Formal commentaries

Written commentaries on the draft guidelines raised several additional major points. On the one hand, the guidelines’ focus on “pragmatic science” was seen as an important complement to traditional experimental clinical science.^6^ They were also seen as a valuable instrument for strengthening the design and conduct of improvement research, potentially leading to greater synergy with improvement practice,^9^ and for increasing the feasibility of combining improvement studies in systematic reviews. On the other hand, the commentaries identified a number of potential difficulties: the draft guidelines were seen as being inattentive to racial and ethnic disparities in care^8^; their IMRaD structure (Introduction, Methods, Results and Discussion) was judged to be incompatible with the reality that improvement interventions, by design, change over time^7^; and there was concern that their use could result in a “dumbing down” of improvement science.^10^

#### Health disparities

In our view, it would not be useful (even if it were possible) to address every relevant content issue in a concise set of guidelines for reporting improvement studies. We do agree, however, that disparities in care are not considered often enough in improvement work, and that improvement initiatives should address this important issue whenever possible. We have therefore specifically cited the issue of care disparities among important outcomes to consider (table 1, item 13(b)(ii)).

#### The IMRaD structure

The Methods sections of scientific reports traditionally describe study protocols that are rigidly fixed, as required by the dictates of experimental design.^19^ Improvement, in contrast, is a “reflexive” learning process—that is, improvement interventions are most effective when they are modified over time in response to outcome feedback. On those grounds, it has been suggested that reporting improvement interventions in the IMRaD format requires multiple sequential Methods sections, one for each iteration of the evolving intervention.^7^ This apparent lack of fit between the realities of improvement practice and the traditional reporting structure for experimental studies has generated considerable debate, which remains unresolved since a reasonable case can be made both for and against use of the IMRaD structure. However, we continue to argue that, as is true for all reports of scholarly inquiry,^20^ reports of improvement studies need to answer A Bradford Hill’s four fundamental questions: Why did you start? What did you do? What did you find? And what does it mean? In our view, that generic requirement justifies using a single Methods section to describe the initial improvement plan and the theory on which it is based; the changes in interventions over time and the learning that comes from making those changes then belong in the Results section rather appearing than in a series of separate Methods sections, since they are themselves important improvement outcomes.^1^

#### “Dumbing down” improvement reports

The main declared purpose of all publication guidelines is to improve the completeness and transparency of reporting. Since it is precisely those characteristics of reporting that make it possible to detect weak, sloppy, or poorly designed studies, it is difficult to understand how use of the draft guidelines might lead to a “dumbing down” of improvement science. The underlying concern here apparently has less to do with transparency, therefore, than with the inference that the draft guidelines were seen as failing to require the rigorous standards of evidence associated with true experimental and quasi-experimental design.^19^ We recognise the importance of those standards in protecting the integrity of outcome measurements, primarily by reducing selection bias^19^ ^21^; those standards, however, fail to take into account the unique purpose and characteristics of the improvement process.

Unlike the “conceptually neat and procedurally unambiguous” interventions—drugs, tests and procedures—whose efficacy is traditionally studied in clinical research, improvement is essentially a social process. Its immediate purpose is to change human performance, and it is driven primarily by experiential learning.^22^ ^23^ It is therefore inherently context dependent and, as noted, reflexive; it is unstable; and it generally involves complex, multi-component interventions. Although traditional experimental and quasi-experimental methods are clearly important for learning *whether* improvement interventions change behaviour, they do not address the crucial pragmatic (or “realist”) questions about improvement: *what* is it about the mechanism of a particular intervention that works, *for whom* and *under what circumstances*?^24^^–^26 Using methods that will simultaneously answer all of these questions is not an easy task, since the experimental and pragmatic approaches can work at cross purposes. The SQUIRE guidelines attempt to maintain an appropriate balance between these two crucial methodologies.

### Consensus meeting of editors and research scholars

With generous support from the Robert Wood Johnson Foundation we undertook a critical appraisal of the draft guidelines at a two-day meeting in April 2007. Thirty people, including clinicians, improvement professionals, epidemiologists, clinical researchers and journal editors attended, several from outside the United States. Before the meeting we sent participants a reading list and a concept paper on the epistemology of improvement. In plenary and small group sessions, participants at the meeting critically discussed and debated the content and wording of every item in the draft guidelines, recommended changes and provided input on plans for dissemination, adoption and future uses of the guidelines. Working from transcribed audiorecordings of all meeting sessions and flip charts listing the key discussion points, a coordinating group (the authors of this paper, with important administrative support from Joy McAvoy) then revised, refined and expanded the draft guidelines.

#### Delphi process

Following the consensus meeting, we circulated sequential revisions of the guidelines for further comments and suggestions in three cycles of a Delphi process. The group involved in that process included the meeting participants plus roughly 20 additional expert consultants. All participants in this process were then asked whether they would be willing to endorse the final consensus version (SQUIRE).

Several features of SQUIRE are worth noting in particular. First, it distinguishes clearly between the practice of improvement (that is, the complex process of planning and implementing improvement interventions) and the evaluation of those interventions (that is, the equally complex process of designing and executing formal studies to assess whether those interventions work, and why they do or do not work). Second, SQUIRE highlights the essential and unique properties of improvement interventions, particularly their social nature, focus on changing performance, context-dependence, complexity, non-linearity, adaptation and reflexiveness. Third, this version specifies both the elements of study design that assess *whether* improvement interventions work (by minimising bias and confounding) and the elements of methods that assess *why* interventions are or are not effective (by marking out contexts and mechanisms of change). And fourth, this version explicitly addresses the ethical dimensions of improvement and improvement studies. Further differences between the initial draft guidelines and the SQUIRE guidelines are provided in table 2.

**Table 2:** Key features of the SQUIRE guidelines that differ from the initial draft

SQUIRE section and item	Added or changed feature of SQUIRE
Title and abstract	Focuses on the accessibility/retrievability of your article
1 Title	Specifies what is meant by improvement, aim of intervention, study methods
2 Abstract	Separated from title; elaborates on abstract format
	
Introduction	Focuses on the rationale of your study
3 Background knowledge	Asks for characteristics of organisations in which the problem occurs
4 Local problem	No change
5 Intended improvement	More specific about improvement aim, plus what triggered the decision to make changes
6 Study question	Distinguishes the study question from the aim of the improvement
	
Methods	Focuses on what you did
7 Ethical issues	Added item: addresses concrete ethical issues rather than administrative ethics review
8 Setting	Highlights context features relevant to why an intervention succeeds
9 Planning the intervention	Requests specifics on intervention components, factors in choice of the intervention, initial plans for implementation
10 Planning the study of the intervention	Added item: separates study of the interventions from the improvement methods themselves; requests specifics on intervention dose and mechanism, study design, issues of internal and external validity
11 Methods of evaluation	Requests specifics on qualitative and quantitative methods; implementation effectiveness, mechanism, primary and secondary outcomes; data quality
12 Analysis	Requests specifics on qualitative and quantitative approaches; appropriateness of the unit of analysis; power
	
Results	Focuses on what you found
13 Outcomes	Includes characteristics of setting relevant to intervention mechanism; requests specifics on success of implementation, strength of association between intervention and outcomes, missing data
	
Discussion	Focuses on what your findings mean
14 Summary	Highlights the study’s strengths
15 Relation to other evidence	Suggests use of summary table of available published evidence
16 Limitations	Requests specifics on maintenance of improvement and on challenges to internal and external validity
17 Interpretation	Expands on the differences between observed and expected results; strength of the data; influence of context factors; modifications that might increase the intervention’s effectiveness; opportunity and actual financial costs
18 Conclusions	No change
	
Other information	Focuses on factors external to the study itself that could affect findings and conclusions
19 Funding	Requests specifics on funding sources and role of funders in conduct of the study

## LIMITATIONS AND QUESTIONS

The SQUIRE guidelines have been characterised as providing both too little and too much information: too little, because they fail to represent adequately the many unique and nuanced issues in the practice of improvement (for example, matching system changes with the type of healthcare problem: simple, complicated or complex),^27^ or the details of experimental and realist study methods^19^ ^21^ ^24^^–^26; too much, because the detail and density of the item descriptions can seem intimidating to authors. Recognising the impossibility of characterising adequately all the essentials of a specific study design or a domain of inquiry in the item descriptions themselves, the developers of several publication guidelines have created accompanying “Explanation and Elaboration” (E & E) documents that provide much of the depth and detail that are missing from the guidelines.^28^^–^30 Building on this concept, the *Medical Journal of Australia* published a series of analytical articles, each devoted to an individual CONSORT guideline item, which were later collected into a book.^31^

That said, we recognise that the SQUIRE item descriptions are significantly more detailed than those of some other publication guidelines. In our view, the complexity of the improvement process, plus the relative unfamiliarity of improvement interventions and of the methods for evaluating them, justifies that level of detail, particularly in light of the enormously diverse backgrounds of people working to improve health care. Moreover, the level of detail in the SQUIRE guidelines closely resembles that in the guidelines for reporting observational studies, which also involve substantial complexities of study design.^32^ To minimise the difficulty of understanding and using the SQUIRE guidelines we do plan, however, to make available a shortened electronic version, accompanied by a glossary of terms that may be unfamiliar to potential users.

## CURRENT AND FUTURE DIRECTIONS

In this supplement, we advance an E & E document that provides the rationale for including the item in SQUIRE, as well as examples of reporting practice for each guideline item, with commentary on strengths and weaknesses of the examples.^33^ To increase the awareness of SQUIRE we are pursuing simultaneous print publication of the present article, as well as editorial commentary on the article, in several journals, along with links to an electronic version, as has been the practice with the public release of other reporting guidelines. We are also promoting the adoption of SQUIRE as journal editorial policy, and its use in peer review and the editorial process.

The SQUIRE website (www.squire-statement.org) will provide an authentic electronic home for the guidelines themselves, a medium for their progressive refinement and an electronic community for authors, students, teachers, reviewers and editors who are interested in the emerging body of knowledge on improvement. Plans for the site include full and short versions of SQUIRE; its accompanying E & E document; commentaries; background readings on the epistemology and science of improvement; a listserve; links to related sites (for example, sites for other publication guidelines, articles that have cited the initial draft guidelines or the SQUIRE version and the like); an edited Wiki section made up of topic areas anchored in SQUIRE; and perhaps a section where authors can post drafts of manuscripts of improvement studies for critical peer review and assessment before journal submission.

Although the primary purpose of SQUIRE is to improve the reporting of original, data-driven improvement studies, we believe the guidelines can also serve useful educational purposes, particularly as a framework for understanding the epistemology of improvement and the methodologies for evaluating improvement work. We believe, similarly, that they can be helpful in planning improvement interventions and studies of those interventions, and therefore plan to support SQUIRE-related educational efforts in those areas.

The value of publication guidelines has been assessed primarily by measuring their impact on the completeness and transparency of relevant publications.^34^ ^35^ Although such studies are difficult, we will encourage and support efforts to evaluate the impact of SQUIRE on the quality of the published improvement literature. And, finally, since publication guidelines are only as strong as their constituent items, we will in addition support efforts to provide empirical evidence that individual guideline items contribute materially to the validity and value of published information in improvement science.

**Summary**

- Published reports of medical quality improvement are relatively few, and reporting is often inadequate.
- The SQUIRE guidelines presented here were developed to encourage more and better reporting of formal improvement studies.
- This paper describes the consensus process used to develop the SQUIRE guidelines.
- The current paper also considers limitations, questions and future directions of the SQUIRE project.

**Linked information**

- The EQUATOR Network. Enhancing the QUality And Transparency Of health Research. Available at http://www.equator-network.org
- The SQUIRE guidelines. Standards for QUality Improvement Reporting Excellence. Available at http://www.squire-statement.org
